# Differences in Nonspecific Low Back Pain between Young Adult Females with and without Lumbar Scoliosis

**DOI:** 10.1155/2019/9758273

**Published:** 2019-03-03

**Authors:** Wangshu Yuan, Jianxiong Shen, Lixia Chen, Hai Wang, Keyi Yu, Hui Cong, Jingya Zhou, Youxi Lin

**Affiliations:** ^1^Department of Rehabilitation and Physical Therapy, Peking Union Medical College Hospital, Beijing, China; ^2^Department of Orthopedic Surgery, Peking Union Medical College Hospital, Beijing, China; ^3^Department of Medical Records & WHO-FIC Collaborating Center, Peking Union Medical College Hospital, Beijing, China

## Abstract

**Study design:**

Retrospective characterization of nonspecific low back pain (NSLBP) in young adult female patients with and without lumbar scoliosis.

**Background:**

There is no consensus as to whether NSLBP in scoliosis patients is related to scoliosis per se or is just a normal symptom that could happen in anyone.

**Objectives:**

The aim of this study was to compare the differences in NSLBP between young adult female patients with and without lumbar scoliosis and to provide a theoretical basis for differential treatment of NSLBP in patients with and without lumbar scoliosis.

**Methods:**

Ninety female young adults with NSLBP were divided into scoliosis and nonscoliosis groups. Characteristics of pain, lumbar mobility, muscle strength, Cobb angle, axial trunk rotation (ATR) angle, and surface electromyography (SEMG) signal were compared between the two groups.

**Results:**

The pain location in scoliotic patients was more concentrated on the left side of the lumbar spine (*P* ≤ 0.001). The area affected by pain (*P*=0.028) and the numerical pain rating scale (NPRS) scores (*P*=0.014) of scoliotic patients were less than those of nonscoliotic patients. The difference between side-bending in scoliotic patients was greater than that in nonscoliotic patients (*P*=0.001). Scoliotic patients exhibited a significantly better ability for flexion (*P*=0.001) and extension (*P*=0.017) than nonscoliotic patients. The posterior muscles in scoliotic patients were stronger than those in nonscoliotic patients (*P*=0.014). The ratio of root-mean-square (RMS) on paraspinal muscles in scoliotic patients was greater than that in nonscoliotic patients (*P* ≤ 0.001). Scoliotic patients exhibited greater relaxation time during the flexion-relaxation phenomenon (FRP) than nonscoliotic patients (*P*=0.024).

**Conclusions:**

The characteristics of NSLBP experienced by patients with lumbar scoliosis were distinct from those of NSLBP experienced by nonscoliotic patients. The treatment of NSLBP in scoliotic patients should be different from that in nonscoliotic patients.

## 1. Introduction

Idiopathic scoliosis is a common spine deformity. A majority of cases of idiopathic scoliosis occur in adolescents, and the disease progression is much faster and greater in adolescents; therefore, adolescent idiopathic scoliosis (AIS) has received more attention than other kinds of idiopathic scoliosis [1]. Owing to the matured skeleton and typically slow rate of disease progression, young adult idiopathic scoliosis (YAIS) has not garnered much attention, either from a clinical or a societal perspective. However, all AIS patients eventually become YAIS patients, and there will be some new cases diagnosed in young adults; therefore, the number of YAIS patients is much greater than that of AIS patients. Generally, YAIS patients with mild disease experience no functional limitation and their aesthetic appearance is also acceptable; therefore, these patients typically consult a doctor because of low back pain (LBP). Research on the relationship between scoliosis and LBP has largely been focused on adolescent people and most cases of AIS experience nonspecific low back pain (NSLBP). Studies have shown that patients with AIS have a higher prevalence of LBP and experience more severe and longer duration of pain as compared to normal controls [2–4]. However, there is no definitive evidence to suggest that scoliosis is indeed the underlying cause of NSLBP in patients with AIS or whether the NSLBP is similar to that occurring in nonscoliotic patients [5, 6]. In this retrospective study, we sought to assess the differences between NSLBP experienced by young adult females with and without scoliosis and investigated the associated factors. Our aim was to provide a theoretical basis for a clearer understanding of NSLBP in young adult patients with scoliosis. To the best of our knowledge, this is the first study that compares the NSLBP experienced by scoliotic and nonscoliotic young adults.

## 2. Materials and Methods

### 2.1. Subjects

All patients were enrolled at the Department of Physical Therapy and Rehabilitation between January 2016 and May 2017, and were diagnosed as NSLBP by orthopedic surgeons. NSLBP has been widely described as pain or discomfort that is localised below the costal margin and above the inferior gluteal folds, with or without leg pain, but not attributable to a known or specific pathology [7]. Inclusion criteria were as follows: (1) female patients diagnosed as NSLBP by both orthopedic surgeons and rehabilitation physicians; (2) age range, 18–30 years; (3) duration of pain: 1–6 months; no history of treatment (physical or pharmacological); (4) patients with idiopathic scoliosis only had a lumbar curve (apex located between L2 and L4), and the Cobb angle was >10°. The exclusion criteria were as follows: (1) LBP caused by any specific reason; (2) history of spine surgery or injury; and (3) patients with intellectual disability and those unable to understand the instructions. A total of 41 patients with scoliosis suffering from NSLBP (mean age: 24.95 ± 2.90 years; mean height: 162.41 ± 3.82 cm; mean body weight: 55.12 ± 7.41 kg) and 49 nonscoliotic patients with NSLBP (mean age: 24.73 ± 2.83 years; mean height: 162.85 ± 3.72 cm; mean body weight: 55.64 ± 6.42 kg) qualified the study selection criteria and were recruited in the study.

There were no significant differences between the two groups with respect to baseline characteristics (Table 1). All patients with scoliosis had undergone radiographic imaging and standard evaluation. The Institution Review Board of the Peking Union Medical Hospital has approved the study and the consent form.

### 2.2. Evaluation and Procedure

#### 2.2.1. Description of Low Back Pain

All subjects were asked to answer the following four questions. First question, where is the location of the pain exactly? The response options were as follows: (1) left side of the lumbar spine; (2) right side of the lumbar spine; (3) middle of the lumbar spine; and (4) both sides of the lumbar spine. Second question, how many square centimeters (cm^2^) of the painful area? The response options were as follows: (1) <5 cm^2^; (2) 5–10 cm^2^; and (3) >10 cm^2^. Third question, how severe is the pain? The subjects were asked to rate the intensity of symptoms on 10-point numerical pain rating scale (NPRS), where 0 indicated no pain and 10 indicated the maximum imaginable pain [8]. Fourth question, does the pain happen suddenly or slowly at the very beginning? The response options were as follows: (1) sudden onset and (2) slow onset.

#### 2.2.2. Mobility of the Lumbar Spine

A physical therapist (PT) evaluated the mobility of the lumbar spine in three planes. (1) In the frontal plane, the subject was made to stand straight against the wall and asked to bend towards the left and right sides to the maximum possible extent. The distance from the middle fingertip to the floor in both standing and the bended position were recorded. The difference between the distance in the standing and side bended position denoted the ability of side-bending. (2) In the sagittal plane, the subject assumed the prone position and pushed her upper body up slowly with her arms, while the PT held her sacrum; the subject was asked to stop moving at the point when the PT felt lifting of her sacrum. The distance of the jugular notch from the bed was recorded to denote the extent of extension. The distance from the middle fingertip to the feet in sit-and-reach was recorded to denote the extent of flexion. (3) In the horizontal plane, the subject held the gymnastics rod on her shoulders and rotated her trunk to both the left and the right side without moving her buttocks in the sitting position. The angle of the rotation of the gymnastics rob was recorded to denote the angle of left and right rotation.

#### 2.2.3. Strength of the Core Muscles around the Lumbar Spine

The strength of the anterior and posterior muscles of the lumbar spine was evaluated by a PT. The subject lifted her legs in the supine position, held the thigh at an angle of 45° to the ground, and the calf kept parallel to the ground. The duration of time was recorded to denote the strength of the anterior muscles of the lumbar spine. Subject was asked to undergo a body weight-dependent isometric back extension test (Sorensen Test, ST) whilst lying on a bed [9]. The time duration was recorded to denote the strength of the posterior muscles of the lumbar spine.

#### 2.2.4. Cobb Angle

The Cobb angle was measured from the anterior-posterior X-rays by recording the angle between the upper and lower most-tilted end vertebra by a spine specialist orthopedist (>20 years experience in scoliosis operative treatment) and a PT (>5 years experience in scoliosis conservative therapy) [10, 11]. The average Cobb angle is 26° degree and the maximum angle is 40°.

#### 2.2.5. Axial Trunk Rotation Angle

The subject was in standing position with 30 cm distance between the feet, and the PT sat behind them. The subjects were asked to bend forward slowly until the point when the deformity of the spine was most prominent and the top of the hump was parallel to the PT's line of sight. The scoliometer was laid across the apical spinous process and the axial trunk rotation (ATR) angle recorded.

#### 2.2.6. Surface Electromyography

The back of the subject was exposed and cleaned with 70% alcohol to reduce skin impedance. For scoliotic patients, the PT palpated the spinous processes, identified the apical spinous process and the intervertebral space between L4 and L5, and marked the skin for placement of electrodes. Six electrodes were placed bilaterally on the marked skin over the erector spinae muscle at the corresponding apical vertebral level and with a 3 cm gap. The other six electrodes were placed bilaterally on the marked skin over the intervertebral space between L4 and L5. For nonscoliotic patients, the electrodes were placed bilaterally on the L2 and L4-L5. Surface electromyography **(**SEMG) signals were recorded during two movements.

*(1) Extension Force*. The subject assumed the sitting position with his back to the wall (distance between the back and the wall was 10–20 cm) and extended the trunk until the occiput touched the wall. At that point, the subject was asked to push the occiput against the wall with as much force as possible for 30 seconds. Values of root-mean-square (RMS; microvolt) as the SEMG signal were collected. In the upper segment (apical spinous process or L2), the greater value was the numerator, and the smaller value was the denominator. The ratio of RMS value on bilateral lumbar was used to assess the difference:

(1) $$\begin{array}{c} \text{ratio of RMS}=\frac{\text{RMS value }\left(\text{greater value}\right)}{\text{RMS value }\left(\text{smaller value}\right)}. \end{array}$$

*(2) FRP* [12]. In the standing position, the subject was asked to bend over to the maximum possible extent on hearing the tone and then to maintain relaxation for 10 s. Subsequently, the subject was asked to straighten up on hearing the tone again; the entire process was performed three times. Values of RMS as the SEMG signal were collected during all the processes. In the lower segment (L4-L5), we recorded the number of times the average RMS value was smaller than 5 mV in the stooped position.

### 2.3. Statistical Analysis

All statistical analyses were conducted using SPSS (version 19.0) software (SPSS, Chicago, IL). The data were analyzed using the chi-squared test, analysis of variance, Student's *t*-test, Spearman correlation coefficient, and linear regression. *P* < 0.05 was considered indicative of statistical significance.

## 3. Results

The distribution of pain in scoliotic patients was largely unilateral. In the scoliosis group, 32 patients had left-sided lumbar pain and 9 patients had right-sided lumbar pain; and 78% of scoliotic subjects had pain on the convex side. In contrast, 83.7% subjects in the nonscoliosis group had midline or symmetrical pain. The painful area in scoliotic patients was smaller than that in the nonscoliotic patients. Nonscoliotic patients had higher NPRS scores than scoliotic patients (Table 2).

The extent of side-bending in the scoliosis group was significantly greater than that in the nonscoliosis group. The extent of flexion and extension in the scoliosis group were significantly greater than that in the nonscoliosis group. Other movements of the lumbar spine were comparable between the two groups (Table 3).

The posterior muscles in scoliotic patients were significantly stronger than those in nonscoliotic patients. No significant between-group difference was observed with respect to the strength of anterior muscles (Table 4).

Scoliotic patients showed significantly greater Cobb angle and ATR angle as compared to nonscoliotic patients (Table 5).

The ratio of RMS value on bilateral lumbar in scoliotic patients was significantly greater than that in nonscoliotic patients. Scoliotic patients showed significantly greater relaxation times in TFRP than in nonscoliotic patients (Table 6).

## 4. Discussion

NSLBP and idiopathic scoliosis are two diseases with unclear etiology; currently, the two conditions can only be distinguished based on symptoms and clinical evaluation. Contrary to the findings reported by Gremeaux et al. and Jackson et al. [13, 14], patients with scoliosis showed lesser severity of pain in the present study. The study by Gremeaux et al. found no significant difference between scoliotic and nonscoliotic patients with regard to pain severity. In the study by Jackson et al., pain severity was greater in scoliotic patients. In the present study, most patients in the scoliosis group had unilateral pain especially on the convex side of the lumbar curve, which is different from the results reported by Joncas et al. [15]. These differences could be attributable to different age profile of the study population. The average age of subjects in these studies was >50 years, while the average age of subjects in the present study was 24 years. Younger subjects are less affected by age-related degenerative changes. It is likely that the pain was more closely associated with the scoliosis at the very beginning; with increase in age, the patients gradually experienced pain from NSLBP. With further progression of age, scoliotic patients experience pain due to both scoliosis and NSLBP; therefore, the pain severity was even greater than that in the control group. The location of pain on the convex side is not just because of the uneven biomechanics caused by vertebral deviation and rotation but may also be related to psychological factors [16, 17]. Most female young adults with scoliosis attach importance to the aesthetic aspects; therefore, the presence of a hump on the lower back is likely to induce regional pain due to psychological reasons.

In the present study, scoliotic patients exhibited uneven side-bending which is different from the results reported by Veldhuizen and Scholten [18]. From a biomechanical perspective, patients with a left lumbar curve experience restriction of bending towards the left side. However, the left lumbar curve is likely to induce trunk deviation to the left side, which facilitates bending towards the left side. This represents a natural compensatory mechanism. Therefore, in the study by Veldhuizen et al., there was no difference between the extent of bending on the two sides. However, the presence of pain, especially unilateral pain, serves to further exaggerate the uneven side-bending. Studies by Hultman et al. [19] and Taechasubamorn et al. [20] showed that patients with low back pain have significantly lesser sagittal flexibility and endurance of back extensors than healthy people. In this study, patients in the scoliosis group showed significantly greater sagittal flexibility and endurance of back extensor muscles as compared to patients in the nonscoliosis group. This suggests that the NSLBP in nonscoliotic patients caused significantly greater disability than in scoliotic patients. In other words, scoliotic patients showed a less stronger relationship with the restricted flexibility and weak muscle strength.

RMS is the time threshold indicator of SEMG; it was shown to reflect the functional capacity of the muscles and was linked to neuromuscular efficiency [21]. In the present study, the RMS value on the convex side of the apical vertebral region was greater than that on the concave side, which is consistent with the results of previous studies [22–25]. During isometric contraction of homologous muscles under the same load, atrophic muscles were shown to produce greater RMS values than fatigued muscles, and fatigued muscles produced greater RMS values than trained muscles [26]. In patients with scoliosis, the convex side muscles generated greater RMS value than the concave side; therefore, we predict that the convex side muscles were weaker than those on the concave side and that the uneven functional capacity of paraspinal muscles was one of the reasons why a significant proportion of patients in the scoliosis group had unilateral pain. FRP was commonly observed in healthy subjects. In contrast, few patients with low back pain demonstrated FRP [27]. In this study, FRP in nonscoliotic patients with NSLBP was less commonly observed than that in scoliotic patients with NSLBP. These findings imply that the FRP in scoliotic patients with NSLBP was more akin to that in healthy subjects.

In conclusion, irrespective of the pain description or pain-related parameters, NSLBP in young adult females with scoliosis is not the same as that in nonscoliotic patients. These findings suggest that the treatment of NSLBP in scoliotic patients should be different from that in nonscoliotic patients.

## Figures and Tables

**Table 1:** Descriptive analysis of the characteristic of subjects in the two groups in this study (SD, standard deviation).

	Scoliosis group	Nonscoliosis group	*P* value	*t* value
Age (years)	24.95 ± 2.90	24.73 ± 2.83	0.721	−0.358
Height (standing) (m)	162.41 ± 3.82	162.85 ± 3.72	0.578	−0.558
Weight (kg)	55.12 ± 7.41	55.64 ± 6.42	0.722	0.357
BMI (kg/m^2^)	20.78 ± 2.66	21.13 ± 2.67	0.538	0.618

**Table 2:** Descriptive statistics of description of the low back pain in two groups.

	Scoliosis group	Nonscoliosis group	*P* value	*Z* value
Pain location	1 (1, 2.5)	3 (3, 4)	≤0.001^*∗*^	−4.316
Painful area	2 (2, 2)	2 (2, 3)	0.028^*∗*^	−2.400
NPRS	3.5 (2.5, 6.0)	5.5 (3.5, 7.5)	0.014^*∗*^	−2.443
Pain onset	2 (1, 2)	1 (1, 2)	0.181	−1.604

^*∗*^Difference between two groups is significant, *P* < 0.05.

**Table 3:** Mobility of the lumbar spine in 3 planes in two groups.

	Scoliosis group	Nonscoliosis group	*P* value	*t*/*Z* value
Left side-bending (cm)	17.73 ± 3.91	17.47 ± 3.67	0.744	−0.328
Right side-bending (cm)	19.41 ± 3.85	18.55 ± 3.56	0.273	−1.013
Difference between side-bending (cm)	3 (1, 5)	1 (1, 2)	0.001^*∗*^	−3.329
Left rotation (°)	57.20 ± 16.43	55.00 ± 18.54	0.558	−0.589
Right rotation (°)	61.95 ± 14.31	57.22 ± 17.82	0.175	−1.368
Difference between rotation (°)	5 (0, 10)	5 (0, 5)	0.103	−1.629
Flexion (cm)	5 (0, 15)	0 (−12.5, 6)	0.001^*∗*^	−3.462
Extension (cm)	18.22 ± 3.34	16.39 ± 3.76	0.017^*∗*^	−2.423

^*∗*^Difference between two groups is significant, *P* < 0.05.

**Table 4:** Descriptive statistics of strength of the core muscles around the lumbar spine in both groups.

	Scoliosis group	Nonscoliosis group	*P* value	*Z* value
Anterior muscle(s)	60 (41, 69)	41 (36, 70)	0.340	−0.955
Posterior muscle(s)	74 (48, 154)	64 (35, 75)	0.014^∗^	−2.469

^*∗*^Difference between two groups is significant, *P* < 0.05.

**Table 5:** Descriptive statistics of Cobb angle and ATR angle in two groups.

	Scoliosis group	Nonscoliosis group	*P* value	*Z* value
Cobb angle (°)	26 (20, 30)	1 (0, 5)	≤0.001^*∗*^	−5.508
ATR angle (°)	8 (7, 9)	2 (0, 2)	≤0.001^*∗*^	−5.555

^*∗*^Difference between two groups is significant, *P* < 0.05.

**Table 6:** SEMG activities in two groups.

	Scoliosis group	Nonscoliosis group	*P* value	*t*/*Z* value
Ratio of RMS	1.59 ± 0.44	1.24 ± 0.40	≤0.001^*∗*^	3.956
Times of FERP	3 (1.5, 3)	2 (0, 3)	0.024^*∗*^	−2.225

^*∗*^Difference between two groups is significant, *P* < 0.05.

## Data Availability

The data used to support the findings of this study are restricted by the Institution Review Board of Peking Union Medical College Hospital in order to protect patient privacy. Data are available from Yuan Wangshu (ywsartemis@126.com) for researchers who meet the criteria for access to confidential data.
